# Acknowledgement to reviewers

**DOI:** 10.1530/EO-4-A1

**Published:** 2024-12-19

The editorial board wishes to thank the following individuals who have helped to review manuscripts for *Endocrine Oncology* during 2023. Their assistance is gratefully acknowledged.

S Adam

A Albani

E K Alexander

M Allinovi

M Araujo-Castro

G Arnaldi

M Asim

I Bancos

N H Bander

M Barbot

R Barroso-Sousa

J-P Bayley

S R Bloom

T Boland

G Boons

M Boscaro

W N Brennen

L A A Brosens

P Burman

O Casar-Borota

R T Casey

G Castoria

O Chabre

B G Challis

H Chen

E Christ

A M Clyne

P Collini

J Crona

M A Czepielewski

P L M Dahia

A Dasari

W W de Herder

L de Mestier

A Delvecchio

T Derlin

G Doherty

M Druce

M R Druce

D J Drucker

C Durante

Y S Elhassan

L Emilsson

A Evers

S Ezzat

M Fani

J Favier

R A Feelders

L Fernandez-Trujillo

J Findling

S M J Fliedner

I Fukuda

M Gessi

M A Gorin

M Gotthardt

G Grani

S Grodski

A B Grossman

B Guerin

O A Hakami

T R Halfdanarson

M C Heinrich

J Hofland

A Ibáñez-Costa

J A M J L Janssen

G A Kaltsas

N Karavitaki

S Kasper

E Kassi

J Kemppainen

C H Kim

C M Kitahara

N Kondo

M Korbonits

M Kroiss

B Lapauw

M-F Lin

X Liu

J Luo

C Lussey

E Maher

L J Martin

F Mauvais-Jarvis

I McEwan

P Miccoli

J Miller

P-L Moreau

M Nagao

L Nieman

I J Nixon

V Njar

F Otsuka

D A Pattison

L G Perez-Rivas

A Peri

A Powlson

V Prasad

A Prinzi

J Ramage

G W Randolph

G Raverot

N S Reed

J Refardt

J K Richer

R P Riechelmann

G Rindi

M Robledo

M Sadar

C Schalin-Jantti

S Sengupta

N Shibata

M Shimabukuro

R Sippel

W Sun

A Tabarin

Y Takahashi

T Temma

D A Tennant

C Thirlwell

T Y N Tong

S Trapp

G Trivellin

S Tsagarakis

M van Hoek

L L T van Hulsteijn

M-C Vantyghem

E Vincent

M Vitale

Z Wang

S M Willerth

S Yamada

K A Zukotynski

